# Germline-dependent gene expression in distant non-gonadal somatic tissues of *Drosophila*

**DOI:** 10.1186/1471-2164-11-346

**Published:** 2010-06-01

**Authors:** Michael J Parisi, Vaijayanti Gupta, David Sturgill, James T Warren, Jean-Marc Jallon, John H Malone, Yu Zhang, Lawrence I Gilbert, Brian Oliver

**Affiliations:** 1 Department of Biology, University of Pennsylvania, Philadelphia, PA, USA; 2 Laboratory of Cellular and Developmental Biology, NIDDK, National Institutes of Health, Department of Health and Human Services, Bethesda MD, USA; 3 Department of Biology, University of North Carolina, Chapel Hill NC, USA; 4 CNPS, UMR UPS-CNRS, Universite de Paris Sud, Orsay, France

## Abstract

**Background:**

*Drosophila *females commit tremendous resources to egg production and males produce some of the longest sperm in the animal kingdom. We know little about the coordinated regulation of gene expression patterns in distant somatic tissues that support the developmental cost of gamete production.

**Results:**

We determined the non-gonadal gene expression patterns of *Drosophila *females and males with or without a germline. Our results show that germline-dependent expression in the non-gonadal soma is extensive. Interestingly, gene expression patterns and hormone titers are consistent with a hormone axis between the gonads and non-gonadal soma.

**Conclusions:**

The germline has a long-range influence on gene expression in the *Drosophila *sexes. We suggest that this is the result of a germline/soma hormonal axis.

## Background

Germline development results in two highly adapted and dimorphic cell types; eggs and sperm [1,2]. In adult *Drosophila*, the majority of sexually dimorphic gene expression occurs within the germline, where it is likely dedicated to gamete production [3-6]. Although much of gamete development in *Drosophila *is gonad autonomous (a combination of the germline and proximal somatic support tissues), gene products expressed in non-gonadal somatic tissues are clearly required to support gamete development by mediating metabolism and behavior [7,8].

There is a clear role of non-gonadal and gonad communication in *Drosophila *and non-Drosophilid females to control egg development. Yolk proteins (Yps) are synthesized in the fat body, exported to the hemolymph and ultimately taken up by developing eggs. *Yp *gene expression is positively correlated with nutritional status [9]. Microarray experiments have shown that expression of oogenic gene batteries are altered by nutrient conditions [10] or application of the hormone 20-Hydroxyecdysone (Ecdysone) [11]. Additionally, neurons in the fly brain that secrete insulin-like peptides regulate germline stem cell division in early oogenesis directly linking egg production with nutrient sensing [12]. In non-Drosophilids, mosquitos produce Yp in response to a blood meal that also implicates hormonal signalling from ecdysone as well as biochemical pathways such as the Target-of-Rapamycin (TOR) pathway (reviewed in [13]).

**Figure 1:**
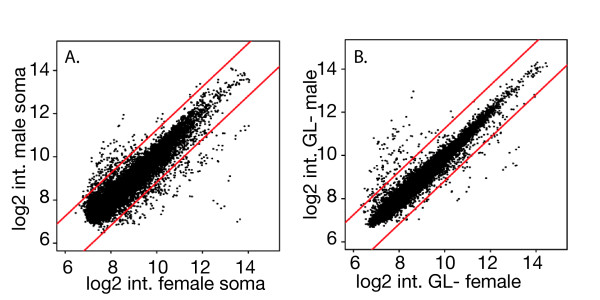
**Log2 intensity (int.) scatterplots show microarray profiles**. A. *y*^*1*^*w*^*67c *^germline-conditioned male vs female soma and B. germline-naïve male vs female whole fly progeny of homozygous *tud*^*1 *^mothers. Elements above and below the red lines indicate differential expression ratios greater than two-fold. Comparison of the two scatterplots shows a reduced number of sex-biased differentially expressed genes in the germline-naive samples.

Expression profiling of females or males with and without germ cells has demonstrated that about half of the sex-biased expression in non-gonadal adult *Drosophila *is germline dependent [6]. This conclusion is based on experiments where wildtype gonadectomized carcasses were compared to non-gonadectomized progeny of *tud*^*1 *^mutant mothers. Despite the absence of somatic gonad structures in the gonadectomized flies, there were more genes with sex-biased expression [6] (Figure 1). This prompted us to directly examine germline-dependent non-gonadal somatic expression at a distance from the germline.

We have extended our previous study [6] to directly compare genotypically matched non-gonadal somatic tissue from flies that developed with a wildtype germline vs. progeny that develop in the absence of a functional germline. We refer to these somas as either germline conditioned or germline naïve (Table 1). Microarray analysis of non-gonadal somatic tissue from germline naïve flies shows batteries of genes that are regulated in a germline-dependent manner. Many of these genes encode metabolic functions consistent with a role in maintaining homeostasis. Interestingly, we find that some genes previously thought to be expressed in a larval-specific, or sex-specific pattern are regulated more indirectly by the presence of germ cells, not by life stage or the sex determination hierarchy. We also found that Ecdysone titers are both highly female-biased and germline dependent. These data are consistent with a *Drosophila *soma/germline hormonal reproductive axis analogous to those found in mammals.

**Table 1:** Pairwise comparisons used for the microarray experiments

Genotype	Sex	Cy3 Germline status	Cy5 Germline status	Notes
*tud*^*1 *^*bw*^*1 *^*sp*^*1*^*/tud*^*1 *^*bw*^*1 *^*sp*^*1*^	male	conditioned	naive	^a^
*tud*^*1 *^*bw*^*1 *^*sp*^*1*^*/tud*^*1 *^*bw*^*1 *^*sp*^*1*^	male	naïve	conditioned	^a^
*tud*^*1 *^*bw*^*1 *^*sp*^*1*^/CyO-DTS	male	conditioned	naïve	^b^
*tud*^*1 *^*bw*^*1 *^*sp*^*1*^/CyO-DTS	male	naïve	conditioned	^b^
*gs(1)*^*N441*^*/Y*	male	conditioned	naïve	^c^
*gs(1)*^*N441*^*/Y*	male	naïve	conditioned	^c^
				
*tud*^*1 *^*bw*^*1 *^*sp*^*1*^*/tud*^*1 *^*bw*^*1 *^*sp*^*1*^	female	conditioned	naïve	^a^
*tud*^*1 *^*bw*^*1 *^*sp*^*1*^*/tud*^*1 *^*bw*^*1 *^*sp*^*1*^	female	naïve	conditioned	^a^
*tud*^*1 *^*bw*^*1 *^*sp*^*1*^/CyO-DTS	female	naïve	conditioned	^b^
*tud*^*1 *^*bw*^*1 *^*sp*^*1*^/CyO-DTS	female	conditioned	naïve	^b^
*tud*^*1 *^*bw*^*1 *^*sp*^*1*^/CyO-DTS	female	conditioned	naïve	^a^
*tud*^*1 *^*bw*^*1 *^*sp*^*1*^/CyO-DTS	female	naïve	conditioned	^a^
*gs(1)*^*N441*^*/gs(1)*^*N441*^	female	conditioned	naïve	^c^
*gs(1)*^*N441*^*/gs(1)*^*N441*^	female	naïve	conditioned	^c^

^a ^Separate cultures for germline-conditioned and naïve flies, allowed to mate.

^b ^Grown in same culture, isolated sexes as virgins and cultured separately for 3-5 days.

^c ^Grown in same culture, allowed to mate.

## Results and Discussion

### Germline-dependent expression in somatic tissue

Given the large energy requirements for gametogenesis, germline naïve flies should have an altered energy balance in the absence of feedback mechanisms. We designed a series of experiments to test for gene expression changes in distant somatic tissues in germline naïve flies. We used maternal effect sterile mutations at two distinct loci for these experiments. The germline forms in embryos by the migration of nuclei into a region of specialized cytoplasm containing germline determinants. Females homozygous for either *gs(1)*^*N441 *^or *tud*^*1 *^[14-17] produce sterile progeny due to defects in the germ plasm or in the migration of nuclei into the germ plasm respectively. Homozygous mutant mothers thus produce viable adults with rudimentary gonads that lack germline cells. These maternal effect mutations allowed us to determine the influence of the germline in genetically identical progeny differing only by the genotype of the mother. Importantly, the mutations affect distinct processes required for germline formation. The only common phenotype is germline ablation. Therefore genes showing expression changes in germline conditioned vs. naïve flies are unlikely to be due to some aspect of the maternal effect unrelated to the germline. It is well known that there is extensive germline/soma communication within the gonad. Because we were interested in studying long-range effects, not local cell-cell communication, we isolated the non-gonadal tissue by removing the full gonads of wildtype and the atrophic gonads of germlineless flies.

**Figure 2:**
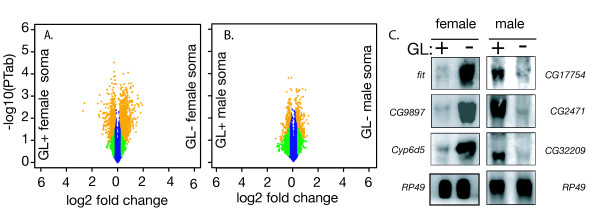
**Volcano plots show ANOVA results**. A. Eight microarrays of female samples for germline-conditioned (GL+) vs -naive (GL-) soma. The y-axis shows log_10_^-x ^*p*-values and the x-axis is log_2 _fold expression. Gene lists are derived from the Fs test in MAANOVA (see Methods) indicated by orange data points. B. Volcano plot of six hybridizations of male germline-conditioned vs -naive soma. Significance and fold expression are as in A. C. Northern blots on genotypically matched (*tud*^*1*^*/CyO) *germline-conditioned (GL+) and naïve (GL-) gonadectomized male and female flies. RP49 serves as a loading control.

To determine if homeostasis is maintained by transcriptional regulation, we performed expression profiling experiments on germline naïve or conditioned somas using the FlyGEM platform [18]. We performed a total of 14 hybridizations comparing germline-conditioned or -naïve flies or of either sex (using flies with the same zygotic genotype, differing only in maternal genotype) and asked what genes show germline-dependent expression (Figure 2 A, B). 169 genes (p < 0.01, F_s _test) were preferentially expressed in the non-gonadal tissues of germline-conditioned females (Additional file 1 Table S1), while 278 genes were preferentially expressed in germline-naïve females (Additional file 2 Table S2). There was slightly less differential expression in males. 60 genes (p < 0.01, F_s _test) were preferentially expressed in the non-gonadal tissues of germline-conditioned males (Additional file 3 Table S3), while 87 genes were preferentially expressed in germline-naïve males (Additional file 4 Table S4). We have previously shown that the FlyGEM results and northern blotting are in good agreement [18]. To validate some of the microarray results showing gene expression is affecting somatic tissue at a distance from the gonad, we performed northern blots on RNA from similarly genotypically matched germline-conditioned and -naive flies. 100% (N = 8) were validated. For example, 7 Cytochrome P450 enzymes are highly expressed in germline-naïve females. Germline-naïve gonadectomized carcasses showed high expression of the Cyp6d5 transcript on northerns as well. The effect of a germline on distant non-gonadal expression was clear (Figure 2C).

To determine the general functions of the genes showing germline dependent expression, we determined enrichment in the Gene Ontology terms [19] using GOstats [20] and a cut-off of p < 0.01 (Fisher's exact test). These data indicate that genes involved in metabolism are differentially regulated in germline-naïve vs germline-conditioned flies (Additional files 5, 6 and 7 Tables S5, S6 and S7). Surprisingly, the germline-naïve flies (both females and males) preferentially express many genes that would be expected to be required for increasing energy utilization. For example, genes encoding serine-type peptidases and amylases were over-represented. This included several members of the '*Jonah' *family of chymotrypsin-like encoding genes that were preferentially expressed in either germline-naïve females (CG7170), germline-naïve males (CG18030, CG10475, CG8867), or both (CG8579). These genes have digestive roles, are expressed in the midgut and have been shown to respond to starvation conditions [10,21,22]. Four triacylglycerol lipases (CG8093, CG6277, CG6271, CG2772) were also preferentially expressed in germline-naïve females. Such lipases would be expected to promote the release of fatty acids for biosynthesis and energy production [23]. Genes encoding proteins with functions in lipid (*lipid storage droplet protein *1 [24]) or protein storage (*larval serum protein 1 *(*Lsp-1*) and *2 *[25]) are also preferentially expressed in germline-naïve flies, suggesting that energy storage is also augmented in the absence of germ cells. Collectively, these data suggest that flies without germ cells have altered expression profiles in pathways required for energy capture and utilization.

### Hormones

Hormone signalling is a possible mechanism by which the germline controls gene expression in distant somatic tissues [8,26]. This view is supported by the expression of genes implicated in hormone biosynthesis. For example, a *Drosophila *Juvenile Hormone esterase (*CG15102*) was preferentially expressed in germline-naïve males suggesting that the effects of germline-conditioning are modulating juvenile hormone metabolism. Also of particular interest are the Cytochrome P450 enzyme genes (*CG17453, CG1944, CG4486, CG11466*) that are preferentially expressed in germline-naïve females. CypP450 proteins are required in the synthesis of a wide range of steroid hormones, including Ecdysone [27].

**Figure 3:**
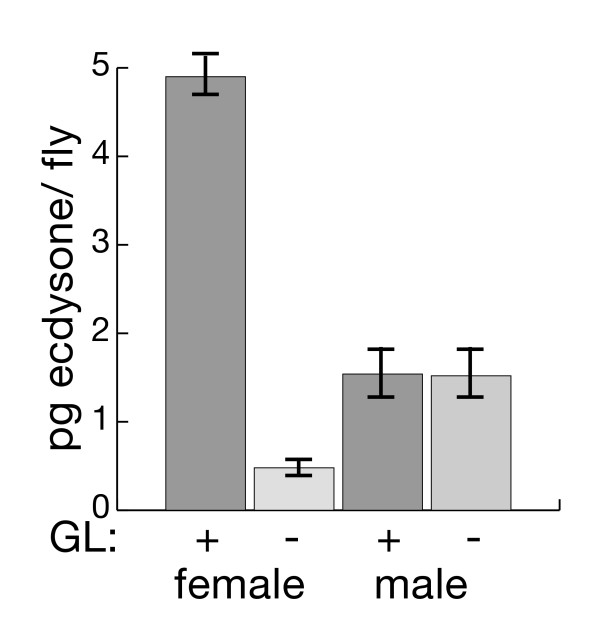
**Ecdysone titers in whole adult flies**. Ecdysone titers averaged from triplicate samples in picograms (pg)/fly were determined by radioimmune assay with the H22 anti-ecdysone antibody.

The best studied *Drosophila *hormone is ecdysone [28]. While we know for example, that mating and insulin signalling alter female ecdysone titers [29,30], with the exception of Handler [31] very little is known about ecdysone titers in adult males. We therefore tested if, and to what extent, ecdysone titers differ between the sexes and if those titers are affected by the germline. Ecdysone radioimmune assays in whole fly extracts from germline-conditioned and -naïve whole flies [32] showed a striking female-bias in ecdysone titers in germline-conditioned flies (Figure 3). We also observed a reduction of ecdysone titers to ~10% of that seen in conditioned females in germline-naïve females. Interestingly, there was no effect of germline on ecdysone titers in males. These results confirm that the bulk of ecdysone produced in the female is germline-dependent, but ecdysone is also produced in males and is not germline-dependent.

### Sex-biased expression due to long-range repression and feedback

**Figure 4:**
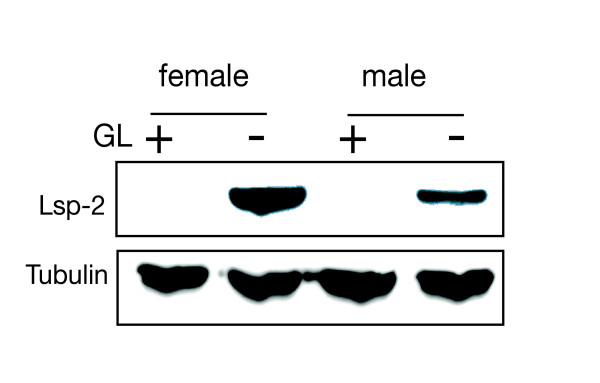
**Western blot analysis on germline conditioned vs naive male and female carcasses using Lsp-2 antisera**. Germline-conditioned (+) and germline-naïve (-) male and female samples show perdurance of Lsp-2 protein in adults. Tubulin serves as a loading control.

Germline-dependency confounds life stage and sex-biased expression patterns: Adult flies are characterized by gamete production, therefore at least some genes showing stage specific expression might do so as a result of the germline and not the developmental stage *per se*. For example, the *Larval serum protein 2 *(*Lsp-2*) gene encodes a hexamerin-like storage protein found throughout the invertebrates that is expressed in the head fat body [25,33]. As suggested by the name, it is highly expressed in third instar larvae and found at low levels in adults [34,35]. We found that *Lsp-2 *mRNA is preferentially expressed in both germline-naïve males and females at the mRNA (Additional files 2 and 4 Tables S2 and S4) and at the protein level (Figure 4). Thus, we suggest that the third instar specific expression in wildtype flies is due at least in part to the absence of maturing gametes in larvae. Our results suggest that *Lsp-2 *expression is de-repressed in the absence of an adult germline. Perhaps, metabolic output normally destined for the egg is shunted to Lsp production in the absence of a germline. Interestingly, the *Lsp-2 *gene is regulated in adult females by ecdysone at the transcriptional level in the head fat body [25,36] supporting the idea that there is a germline-dependent hormonal axis.

We also asked if some sex-biased expression in *Drosophila *is due to repression by the germline of the opposite sex. Three genes (*Sodh-1, CG5288 *and *CG11236*) with highly male-biased expression in the head [37] were preferentially expressed in germline-naïve females. Similarly, two of the *Cyp450 *genes preferentially expressed in germline-naïve females (*Cyp12a4 *and *Cyp309a1*) show highly male-biased expression in wildtype [38]. This suggests that some genes with male-biased expression are repressed by a female germline. Interestingly, expression patterns of two of these genes with male-biased expression in wildtype flies, and high expression in germline-naïve females, Sodh-1 and CG5288, show increased expression when *timeless *expressing neurons are perturbed [39], raising the possibility of a germline CNS interaction.

We also found evidence for negative feedback on sex-biased gene expression. The *female sterile independent of transformer *(*fit*) gene is expressed in the female head fat body [37] and showed even more dramatic female-biased expression in germline-naïve females in our experiments. Thus, the germline may also regulate feedback circuits to dampen sex-biased expression. These findings have important implications for ongoing work to determine the targets of somatic sex determination genes [4,40]. We had previously observed that at least half of the sex-biased expression in the soma is due to the germline [6], suggesting that long range germline-dependent expression may be as important as the sex determination hierarchy in many aspects of sex-biased expression. Because the mutants used to probe the sex determination hierarchy (such as females transformed to males by *transformer *or *doublesex *alleles, or males transformed to females by ectopic expression of *transformer*) often have severe effects on germline development [41], it is not possible to determine if sex-biased expression in those animals is due to autonomous action of the hierarchy or an indirect effect of altered germline development from expression profiles in these mutants by simple profiling experiments.

### Mating behavior

**Figure 5:**
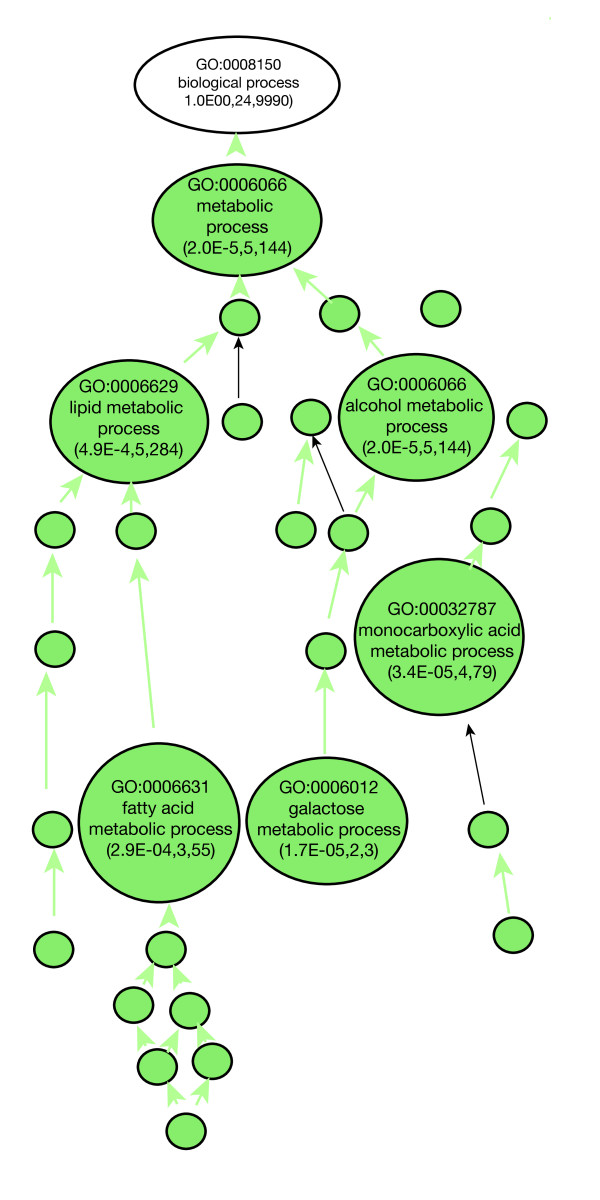
**GO terms represented in genes that upregulated in both germline-naive and unmated females**. The large circles are expanded nodes that show text indicating GO IDs, GO term and score for that node. The score includes the *p*-value, number of genes in the query set annotated to that node and the number of genes in the database annotated. Smaller circles indicate the collapsed nodes that meet the pruning threshold, but not the collapsing threshold.

We observed that 78 of the genes showing reduced expression in germline conditioned females relative to germline naïve females were among the genes showing reduced expression in mated females relative to unmated females [7]. Direct comparisons of data sets between the studies is complicated by differences in data handling and experimental designs. However, if there is a relationship between mating and germline presence, then a coherent set of functions should be implicated by both studies. To investigate this connection further, we uploaded the lists of genes regulated by sperm, seminal fluid, or seminal fluid components [7] and the lists of genes regulated by the germline and merged intersected IDs in the data mining environment at Flymine [42] and visualized the Gene Ontology term hierarchy in VLAD [43]. These genes that are negatively regulated by the germline and by mating are enriched for lipid and carbohydrate metabolism functions (Figure 5).

To more directly assay for similarities in expression profiles in unmated germline conditioned females and in germline naïve females, we performed two hybridizations of mRNA prepared from the carcasses of unmated germline naïve females and unmated germline conditioned females (Table 1). We found no significant differences (FDR corrected p < 0.1, t-test). These preliminary results are consistent with a non-additive effect of mating and germline conditioning, suggesting that both the mating response and the germline conditioning response are in the same pathway. It is unclear if germline dependent expression is caused by failure to mate. We found that germline naïve females resisted mating in pair-mating experiments in 1 hour bouts, but we observed sperm transfer in longer mating assays (data not shown). Additionally, germline naïve females have been used in previous studies and no mating latency was observed ([44] reviewed in [45]).

## Conclusions

In conclusion, although much of sex determination and differentiation in the *Drosophila *soma is cell autonomous, our data show a surprising amount of sex-biased expression in the soma is germline dependent. In mammals, gene expression in distant tissues and organs is coordinated by sex hormones produced in the gonads. It seems likely that a thematically similar hormonal axis exists in *Drosophila*.

## Methods

### *Drosophila *strains and culture

*Drosophila *strains were cultured on standard cornmeal medium (Tucson Drosophila Stock Center, University of Arizona, Tucson). Descriptions of strains and specific alleles can be found at FlyBase [46]. To isolate germline-conditioned flies, heterozygous *tud*^*1*^*bw*^*1*^*sp1*^*1*^*/CyO *or *gs(1)*^*N441*^*/FM7 *females were crossed to *tud*^*1*^*bw*^*1*^*sp*^*1*^*/CyO *or *gs(1)*^*N441*^*/Y *males respectively which gave germline-conditioned homozygous mutant females. To generate germlineless progeny, homozygous females were crossed to *tud*^*1*^*bw*^*1*^*sp*^*1*^*/CyO *or *gs(1)*^*N441*^*/Y *males respectively. All progeny from these mutant mothers were germlineless. Microarray hybridizations were performed with genotypically matched progeny of these crosses (Table 1). These progeny were aged 5-7 days after eclosion, gonadectomized and their carcasses frozen on dry ice. To test culture conditions, 2 *tud*^*1*^*bw*^*1*^*sp1/CyO *heterozygous and 5 homozygous *tud*^*1*^*bw*^*1*^*sp*^*1 *^*or gs(1)*^*N441 *^mothers were crossed to males within the same vial. These produced both germline-conditioned and -naive progeny in the same culture vial. We also included a set of arrays on virgin flies to test mating differences as a variable (see [7]). Germline development in all cases was determined by examining gonad morphology during dissection.

Similarly, in the case of the mating experiments, we used *tud*^*1 *^and *gs(1)*^*N26 *^to generate genotypically matched progeny with and without a germline. Virgin females and males were collected following eclosion (twice a day). Canton S males were also used in some experiments as indicated. Single males and females were aged for 5 days in glass vials. Unanaesthetized females were introduced into clean glass mating arenas and unanaesthetized males were introduced and observed for at least 45 min at 23-25°C as described in [47]. Females were dissected following the termination of the mating experiments

### Microarrays and data analysis

Somatic tissues from frozen carcasses (full and atrophic gonads were removed) were used to make total RNA using Trizol reagent (Invitrogen, Carlsbad, CA) followed by one round of poly(A) selection with Oligotex (Qiagen, Valencia, CA). Cy3 and Cy5 labeling of the poly(A) RNA was performed for hybridization to FlyGEM microarrays (Incyte Genomics, Palo Alto, CA) as described [18]. Hybridized microarrays were scanned on an Axon 4000A scanner and data was processed with Genepix v5.1 (Molecular Devices, Sunnyvale, CA). Data are available at Gene Expression Omnibus [48,49] under series accession (GSE11017). Microarray data from these and previously published experiments [5,6] were adjusted through a printtip lowess correction followed by quantile cross-normalization across all arrays using the Limma package in Bioconductor [50]. Genes with significantly different expression based on germline status were determined using the ANOVA model of Wolfinger [51] in the R/MAANOVA package ver. 0.96 [52]. The model for gene specific effects according to [52] is:(1)

*Here, μ *is the gene mean; *Ai *(*i *= 1, . . 10) is the array effect; *Dj *(*j *= 1, 2) is the dye effect;

*Sk(i, j) *(*k *= 1, ..., 5) is the sample effect. Here *_i j *is the residual, terms * μ*, *Dj *and *Sk(i, j) *are treated as fixed while term *Ai *is treated as random. We used a mixed model ANOVA with 'Germline' as the sample effect term. Volcano plots (Figure 2) show the results of three F tests from MAANOVA. colored as blue (F_1_), green (F_3_) and orange (F_s_). Gene lists were derived from the F_s _test (for shrinkage estimator), which makes no a priori assumptions about distribution of variances across genes [52] We applied a false discovery rate (FDR) adjustment to the F_s _test results [53] using the default method in MAANOVA. Data analysis and graphics were done with packages in Bioconductor and Gene Ontology [54] analysis was performed with GOstat [55]. Go terms were visualized as a network in VLAD [43]. The node pruning threshold was 2 and the collapsing threshold for annotation of the nodes was set at 3.

### Northern and western blots

Northern blots were performed on genotypically matched (*tud*^*1*^*bw*^*1*^*sp1*^*1*^*/CyO*) gonadectomized germline-conditioned and -naive males and females to corroborate the microarray experiments. Total RNA was isolated with Trizol reagent (Invitrogen, Carlsbad, CA) and separated on denaturing agarose gel electrophoresis [56]. Separated RNA was transferred to Hybond-N+ membrane (GE Healthcare, Piscataway, NJ) and probed with ^32^P labeled amplimers for the genes being tested using FlyGEM platform primer sets [18]. DNA probes were random prime labeled with the Rediprime II labeling system (GE Healthcare, Piscataway, NJ). Hybridization was done at 68° with Ultrahyb (Stratagene, La Jolla, CA) and blots were imaged on a Storm 860 Phosphorimager using Imagequant software (Molecular Dynamics, Sunnyvale, CA) or a Fuji FLA-5000 Phosphorimager and processed with Multi Gauge software (Fujifilm Medical Systems, Stamford, CT).

Western blots were also performed on genotypically matched *tud*^*1*^*bw*^*1*^*sp1*^*1*^*/CyO *gonadectomized carcasses isolated from flies reared as described above. Carcasses were ground in 2× SDS loading buffer (National Diagnostics, Atlanta, GA); 0.5 M Tris-HCl (pH 6.8), 4.4% (w/v) SDS, 20% (v/v) glycerol, 2% (v/v) 2-mercaptoethanol, and bromophenol blue), electrophoresed and transferred using Nupage precast gels and the X-Cell transfer system (Invitrogen, Carlsbad, CA). Rabbit anti*-Drosophila *Lsp-2 and mouse anti-Tubulin antisera were diluted 1:10,000 in 1× PBS, 5% nonfat dry milk. Western signal was detected with Pierce ECL western blotting substrate (Thermo Fisher Scientific, Waltham, MA) using appropriate HRP conjugated secondary antibodies.

#### Radioimmune assays

Measurement of ecdysone titers in whole flies was performed essentially as described [32]. To prepare samples, whole 5-7 day *tud*^*1*^*bw*^*1*^*sp1*^*1*^*/CyO *flies (males and females, germline conditioned and -naïve) were collected and flash frozen on dry ice. Flies were weighed to determine the approximate number contained in each tube and stored at -80°C. Fly weight was determined by collecting flies of the four conditions and weighing them on an Ohaus Galaxy 200D analytical balance (Ohaus, Pine Brook, NJ). There was little difference in the weights of germline-naive compared to germline-conditioned females. 100 germline-conditioned females weighed 0.0974 grams and 100 germline-naive females weighed 0.111 grams. Males weighed 0.0765 and 0.0774 grams/100 flies for germline-conditioned and-naive flies respectively. Ecdysone was extracted by grinding flies in 500 μl methanol 5 min. followed by vortexing 5 hrs., 4°C. Supernatant was removed and frozen and the tissues were back extracted with an additional 500 μl methanol followed by extraction with 250 μl ethanol. The extracts were combined and centrifuged 15 min., 4°C to remove solid debris. Extracts were then dried in a SpeedVac and resuspended in 100 μl H_2_O. Samples were combined with 3H-ecdysone (Amersham, Piscataway), the H22 anti-ecdysone antibody and *Staphylococcus aureus *Protein A (Sigma-Aldrich, St. Louis) and incubated overnight, 4°C. The protein A was pelleted by centrifugation at 13,000 RPM, 4°C and resuspended in 100 μl H_2_O plus 450 μl Cytoscint (Thermo Fisher Scientific, Waltham, MA). Radioimmune assays were run against a set of ecdysone standards ranging in concentration from 2 ng to 15.6 pg and counted in a Perkin Elmer scintillation counter. The ecdysone titer was determined by how much the sample displaced the ^3^H-labeled ecdysone in the reaction.

## Authors' contributions

MJP carried out the Drosophila genetics and sample preparation, microarrays Northern and Western blots, study design and drafted the manuscript. VG performed microarrays and participated in data analysis, DS YZ and JHM participated in data and statistical analysis, JTW, LIG and MJP performed the radioimmune assay, J-M J and BO performed mating assays and BO participated in data analysis, experimental design and coordinated the study. All authors read and approved the final manuscript.

## Supplementary Material

Additional file 1 **Genes preferentially expressed in germline-conditioned vs germline-naïve females**. This file includes gene name, common gene symbol, a hyperlink to FlyBase ID and GO IDs and terms associated with each gene. Preferential expression is shown by *p *values derived from MAANOVA and fold change in expression levels between germline-conditioned vs -naïve females. Click here for file

Additional file 2 **Genes preferentially expressed in germline- naïve vs germline- conditioned females**. This file includes gene name, common gene symbol, a hyperlink to FlyBase ID and GO IDs and terms associated with each gene. Preferential expression is shown by *p *values derived from MAANOVA and fold change in expression levels between germline- naïve vs - conditioned females. Click here for file

Additional file 3 **Genes preferentially expressed in germline-conditioned vs germline-naïve males**. This file includes gene name, common gene symbol, a hyperlink to FlyBase ID and GO IDs and terms associated with each gene. Preferential expression is shown by *p *values derived from MAANOVA and fold change in expression levels between germline-conditioned vs -naïve males. Click here for file

Additional file 4 **Genes preferentially expressed in germline- naïve vs germline- conditioned males**. This file includes gene name, common gene symbol, a hyperlink to FlyBase ID and GO IDs and terms associated with each gene. Preferential expression is shown by *p *values derived from MAANOVA and fold change in expression levels between germline- naïve vs - conditioned males. Click here for file

Additional file 5 **GO IDs and Terms over-represented in germline-conditioned females**. This file includes GO terms over-represented, *p *value resulting from Fisher's Exact Test, common gene symbols associated with the term and number of genes per total number for each term Click here for file

Additional file 6 **GO IDs and Terms over-represented in germline-naive females**. This file includes GO terms over-represented, *p *value resulting from Fisher's Exact Test, common gene symbols associated with the term and number of genes per total number for each term Click here for file

Additional file 7 **GO IDs and Terms over-represented in germline-naive males**. This file includes GO terms over-represented, *p *value resulting from Fisher's Exact Test, common gene symbols associated with the term and number of genes per total number for each term Click here for file
